# Integrating Machine Learning with MALDI-TOF Mass Spectrometry for Rapid and Accurate Antimicrobial Resistance Detection in Clinical Pathogens

**DOI:** 10.3390/ijms26031140

**Published:** 2025-01-28

**Authors:** Xaviera A. López-Cortés, José M. Manríquez-Troncoso, Alejandra Yáñez Sepúlveda, Patricio Suazo Soto

**Affiliations:** 1 Departamento de Computación e Industrias, Facultad de Ciencias de la Ingeniería, Universidad Católica del Maule, Talca 3460000, Chile; manriquez.josematias@gmail.com (J.M.M.-T.); alejandratania9@gmail.com (A.Y.S.); 2 Centro de Innovación en Ingeniería Aplicada (CIIA), Universidad Católica del Maule, Talca 3460000, Chile; 3 Departamento de Microbiología, Facultad de Ciencias de la Salud, Universidad de Talca, Campus Talca, Avda. Lircay s/n, Talca 3460000, Chile; psuazosoto@gmail.com; 4 Centro de Ecología Integrativa, Universidad de Talca, Campus Talca, Avda. Lircay s/n, Talca 3460000, Chile

**Keywords:** antibiotic resistance, *Staphylococcus aureus*, *Escherichia coli*, *Klebsiella pneumoniae*, machine learning, transfer learning

## Abstract

Antimicrobial resistance (AMR) is one of the most pressing public health challenges of the 21st century. This study aims to evaluate the efficacy of mass spectral data generated by VITEK^®^ MS instruments for predicting antibiotic resistance in *Staphylococcus aureus*, *Escherichia coli*, and *Klebsiella pneumoniae* using machine learning algorithms. Additionally, the potential of pre-trained models was assessed through transfer learning analysis. A dataset comprising 2229 mass spectra was collected, and classification algorithms, including Support Vector Machines, Random Forest, Logistic Regression, and CatBoost, were applied to predict resistance. CatBoost demonstrated a clear advantage over the other models, effectively handling complex non-linear relationships within the spectra and achieving an AUROC of 0.91 and an F1 score of 0.78 for *E. coli*. In contrast, transfer learning yielded suboptimal results. These findings highlight the potential of gradient-boosting techniques to enhance resistance prediction, particularly with data from less conventional platforms like VITEK^®^ MS. Furthermore, the identification of specific biomarkers using SHAP values indicates promising potential for clinical applications in early diagnosis. Future efforts focused on standardizing data and refining algorithms could expand the utility of these approaches across diverse clinical environments, supporting the global fight against AMR.

## 1. Introduction

The alarming increase in antibiotic-resistant bacterial strains has led the World Health Organization (WHO) to declare this issue one of the most serious health challenges of the 21st century, with an estimated 4.95 million deaths annually associated around the world [1]. In the United States, this problem is responsible for more than 23,000 deaths annually [2]. Projections indicate that, by 2050, it will cause over 10 million deaths globally, according to the WHO. In Chile, this problem is also on the rise. Data from the Instituto de Salud Pública (ISP) indicate a significant increase in resistant bacteria over recent years [3]. For example, resistance to oxacillin in *Staphylococcus aureus* increased from 37.5% in 2017 to 43.3% in 2020, establishing it as one of the leading causes of surgical wound and bloodstream infections. Similarly, resistance to ciprofloxacin and cefotaxime in *Klebsiella pneumoniae* increased significantly, reaching 73.9% and 74%, respectively, in 2020 [4]. Recent studies have identified key contributors to this crisis, including the misuse of antibiotics in both medicine and agriculture [5,6], as well as environmental factors that facilitate the dissemination of resistance genes [7,8].

In current clinical practice, antibiotic resistance is primarily determined using methods such as antimicrobial susceptibility testing (AST). However, these culture-based methods require 24 to 72 h to produce results, compelling clinicians to prescribe empirical or broad-spectrum treatments in urgent cases. This underscores the need to develop novel methodologies that enable the faster and more accurate detection of antibiotic resistance.

The problem of antimicrobial resistance has been addressed from various perspectives, including the development of new antibiotics [9], the implementation of biochemical strategies to mitigate resistance, and the enhancement of the efficacy of existing antibiotics [10]. In recent years, machine learning (ML) has emerged as a crucial tool in addressing this public health crisis [11,12]. For instance, graph neural networks have been used to identify new chemical compounds with antibiotic potential against methicillin-resistant *Staphylococcus aureus* (MRSA) [13]. Furthermore, bacterial genome sequencing has enabled the development of machine learning models to identify strains resistant to specific antibiotics [14,15].

One technique that has garnered significant interest due to its speed, accuracy, and cost-effectiveness is MALDI-TOF mass spectrometry (Matrix-Assisted Laser Desorption/Ionization Time-Of-Flight) [16]. The protein profile generated by this technique enables clear differentiation between bacterial species [17,18], including the distinction between resistant and susceptible strains to specific antibiotics [19,20]. The integration of this technique with machine learning models has shown great promise as a powerful tool to enhance clinical decision-making in antibiotic treatments [21].

**Figure 1:**
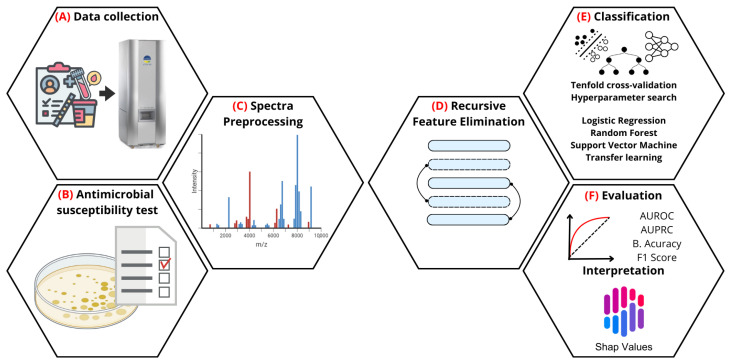
Antimicrobial resistance prediction workflow based on MALDI-TOF mass spectrometry. (**A**) Data collection: bacterial mass spectra are acquired using the MALDI-TOF VITEK MS (BioMérieux). (**B**) Antimicrobial susceptibility testing: bacterial resistance profiles are determined through laboratory assays. (**C**) Spectral pre-processing: data are normalized, and the most relevant peaks between 2000 and 10,000 Da are selected. (**D**) Recursive feature elimination: dimensionality is reduced by selecting the most informative peaks for classification. (**E**) Classification: performed using tenfold cross-validation and hyperparameter tuning with models such as Logistic Regression, Random Forests, Support Vector Machines, and transfer learning. (**F**) Evaluation: metrics include AUROC, AUPRC, balanced accuracy, and F1 score. Results are further interpreted using SHAP values to identify key spectral peaks contributing to antimicrobial resistance prediction.

This study proposes an innovative approach to predict antimicrobial resistance in *Staphylococcus aureus*, *Escherichia coli*, and *Klebsiella pneumoniae*, which the WHO prioritizes. Mass spectra were obtained using MALDI-TOF VITEK^®^ MS instruments. Then, various machine learning algorithms, including Support Vector Machines, Random Forest, Logistic Regression, and CatBoost, were applied to identify specific resistance patterns. A notable innovation of this work is the evaluation of transfer learning (TL) to integrate data from different mass spectrometry instruments, enabling the adaptation of models across diverse platforms. This approach represents a significant advancement, as it could facilitate the deployment of robust models in multiple clinical settings, improving the precision and applicability of predictive diagnostic tools in the fight against antimicrobial resistance. Figure 1 summarizes the workflow of the applied methodology.

## 2. Results

### 2.1. Database

**Figure 2:**
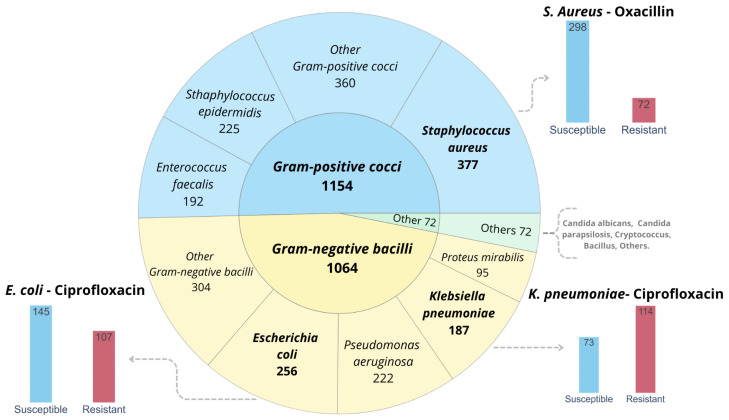
Distribution of bacterial species and their antibiotic susceptibility profile. The central pie chart shows the classification of 2229 clinical isolates into Gram-positive and Gram-negative bacteria. *Escherichia coli* (*n* = 256), *Klebsiella pneumoniae* (*n* = 187), and *Staphylococcus aureus* (*n* = 377), among other bacterial species, are the main pathogens studied. The susceptibility and resistance profile for *E. coli* and *K. pneumoniae* to ciprofloxacin and for *S. aureus* to oxacillin are shown in the graphs in the sidebar. Of the *E. coli* samples, 145 were susceptible and 107 were resistant to ciprofloxacin, while, for *K. pneumoniae*, 73 samples were susceptible and 114 were resistant to the same antibiotic. For *S. aureus*, 298 samples were susceptible and 72 were resistant to oxacillin. Differences in the total number of isolates and susceptibility testing are due to the fact that some samples were not tested against the antibiotics mentioned.

Between September 2022 and July 2023, a total of 2229 MALDI-TOF mass spectra of various bacterial and fungal species were collected. Among the species identified, the most representative group was that of Gram-positive cocci, with a total of more than 1045 samples. Within this group, *Staphylococcus aureus* stood out with 377 samples, followed by *Staphylococcus epidermidis* with 225 samples and *Enterococcus faecalis* with 192 samples. The second predominant group was Gram-negative bacilli, where *Escherichia coli* (256 samples), *Pseudomonas aeruginosa* (222), and *Klebsiella pneumoniae* (187) were identified. Figure 2 gives an overview of the set of bacteria isolated in the Regional Hospital of Talca, as well as a description of the prevalence of resistant strains in the main species of interest.

### 2.2. Classification Models

Table 1 shows the results obtained by the different algorithms implemented in this study, reporting the mean and standard deviation from the 10-fold cross-validation.

In detail, for *S. aureus* resistant to oxacillin, the CatBoost algorithm showed the best performance metrics compared to the RF, LR, SVM, and transfer learning models. CatBoost achieved an AUROC of 0.86, an AUPRC of 0.73, a balanced accuracy of 0.77, and an F1 score of 0.61 (Table 1). This establishes it as the most robust model in terms of both discrimination and accuracy. The RF algorithm ranked second with metrics of AUROC equal to 0.86, AUPRC of 0.68, balanced accuracy of 0.74, and F1 score of 0.56. Additionally, the SVM and transfer learning models underperformed, yielding the lowest metrics, with AUROCs below 0.8 and F1 scores below 0.4 (Table 1).

For the bacterium *E. coli* resistant to ciprofloxacin, the performance of CatBoost was superior, achieving excellent values of 0.91 and 0.91 in AUROC and AUPRC, respectively, as well as a balanced accuracy of 0.81 and an F1 score of 0.78 (Table 1). In contrast, the worst performance was obtained by Logistic Regression, which reached values of 0.68 in AUROC, 0.66 in AUPRC, and 0.64 in balanced accuracy. The SVM and RF models showed AUROC performances equal to 0.77 and 0.76, respectively. Furthermore, SVM has the lowest F1 score at 0.58, indicating its reduced ability to balance precision and recall, whereas RF achieved an F1 score of 0.63. On the other hand, the TL model showed poor performance, with an AUROC of 0.70 and an AUPRC of 0.73 (Table 1).

Finally, in the case of *K. pneumoniae* resistant to ciprofloxacin, the performance of the models was lower compared to *S. aureus* and *E. coli* for AUROC, AUPRC, and balanced accuracy (Table 1). Nevertheless, the CatBoost model outperformed the others, achieving an AUROC of 0.73 and an AUPRC of 0.83. This model also showed an F1 score of 0.78, the highest among all models and species evaluated. Similar to the previous models for *S. aureus* and *E. coli*, the use of transfer learning yielded the poorest performance (Table 1).

Furthermore, in terms of applying the SMOTE technique to this type of data, all models exhibited poor performance across all evaluated metrics (Table S1 in the Supplementary Materials). Specifically, for *S. aureus* and *E. coli*, the CatBoost model outperformed all metrics compared to the rest of the algorithms. Moreover, for *K. pneumoniae*, the best model corresponded to RF. In particular, for *S. aureus* resistant to oxacillin, the CatBoost model achieved AUROC and AUPRC values of 0.80 and 0.65, respectively. In the case of *E. coli* resistant to ciprofloxacin, the CatBoost model gave metrics of 0.77 in AUROC and AUPRC. In regards to *K. pneumoniae* resistant to ciprofloxacin, the RF model yielded AUROC and AUPRC values of 0.68 and 0.77, respectively (Table S1 in the Supplementary Materials).

### 2.3. Analysis of Significant Peaks by Using SHAP Values

**Figure 3:**
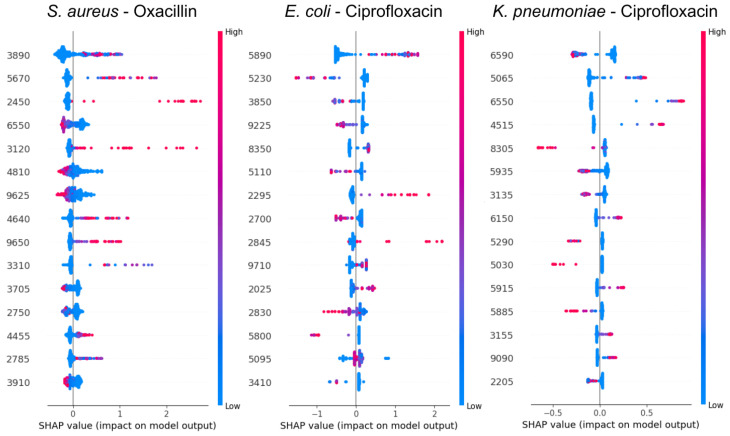
Plots of SHAP values for the major spectral peaks associated with antimicrobial resistance in *Staphylococcus aureus* (oxacillin), *Escherichia coli* (ciprofloxacin), and *Klebsiella pneumoniae* (ciprofloxacin). The plots show the contribution of each spectral peak to the model prediction, where blue dots represent low values of the feature (*m/z* peak) and red dots represent high values. The SHAP values (on the horizontal axis) indicate the impact of a particular peak on the model output: A positive SHAP value indicates that the feature increases the likelihood of resistance, while a negative SHAP value indicates that the feature favors susceptibility. The scatter of the points along the horizontal axis reflects the variability in the effect of each peak in different samples. Thus, peaks with a higher spread and amplitude of SHAP values have a greater influence on the model predictions. This visualization allows the identification of the most relevant spectral biomarkers for the classification of antimicrobial resistance in the different bacterial species under study.

In order to evaluate the feature contribution, the model with the best performance for each bacterium under study was selected. Specifically, the CatBoost models were used to apply Shapley value analysis for the resistant study in *S. aureus*–oxacillin, *E. coli*–ciprofloxacin, and *K. pneumoniae*–ciprofloxacin. Figure 3 shows the Shapley values of the 15 features with the highest average contribution for each of the three cases studied. In the case of *S. aureus*–oxacillin, the ions of 3890, 5670, and 2450 Da play a crucial role in the resistance prediction. In addition, concerning the red color, which indicates a high value of the sample in a certain characteristic, we can see that the presence of ions at 3890, 5670, 2450, 3120, 4640, and 9650 Da have a direct relationship with the prediction of the positive (resistant) class. Moreover, for *E. coli*–ciprofloxacin, the ion at 5890 Da showed a strong association with predicting the resistant class, while the presence of three *m/z* peaks at 5230, 3850, and 9225 were correlated with the prediction of the susceptible class (Figure 3). It is also important to note that, in the case of *E. coli*, from the 15 *m/z* peaks reported, the majority are in the range of 2000–3000 Da, which corresponded to ions of 2025, 2295, 2700, 2830, and 2845 Da, with additional peaks in the 5000–6000 Da range, including ions at 5095, 5110, 5230, 5800, and 5890 Da. Finally, for *K. pneumoniae*–ciprofloxacin, we can see that the absence of the *m/z* 6590 peak is cataloged as the most-determinant factor in the prediction of resistant samples, followed by the presence of the *m/z* peaks 5065, 6550, and 4515 Da. It is also important to note that most of the reported ions were concentrated in the range of 5000–7000 Da, including 5030, 5065, 5290, 5885, 5915, 5935, 6150, 6550, and 6590 Da (Figure 3).

Furthermore, a search was conducted in the UniProt database to identify the most-likely proteins associated with the top-five *m/z* peaks identified as most relevant through the SHAP analysis for each case studied (Figure 3). Table 2 presents the most probable protein biomarker assignments for each of these peaks, highlighting potential correlations with key protein functions and their relationship to antibiotic resistance in the evaluated microorganisms.

## 3. Materials and Methods

### 3.1. Data Acquisition

Between September 2022 and July 2023, antimicrobial susceptibility testing (AST) was performed on a number of samples, resulting in the creation of eight different databases. The process of sample acquisition, characterization, and antibiotic susceptibility testing is detailed below.

#### 3.1.1. Sample Acquisition and Characterization

The analyzed samples of *Escherichia coli*, *Klebsiella pneumoniae*, *Staphylococcus aureus*, and other isolates were obtained from the Regional Hospital of Talca. Samples were cultured on commercial media (Columbia Agar and MacConkey Agar, VALTEK, Santiago, Chile) and incubated at 37 °C for 24 h. Bacterial colonies were then collected, and species identification was performed using the VITEK^®^ MS mass spectrometer (Biomerieux, Paris, France).

#### 3.1.2. Antimicrobial Susceptibility Testing

Antimicrobial susceptibility testing was performed using the disk diffusion method (Kirby and Bauer) on Muller Hinton II plates (VALTEK, Santiago, Chile). Each bacterial colony was adjusted to a 0.5 McFarland standard and exposed to various antibiotics, including ampicillin (10 µg), ampicillin/sulbactam (10/10 µg), ceftazidime (30 µg), ciprofloxacin (5 µg), clindamycin (2 µg), erythromycin (15 µg), ertapenem (10 µg), gentamicin (10 µg), imipenem (10 µg), meropenem (10 µg), oxacillin (30 µg, with cefoxitin substituted for oxacillin), piperacillin/tazobactam (100/10 µg), penicillin (10 units), and vancomycin (30 µg). The plates were incubated at 37 °C for 24 h. Results were evaluated visually by measuring the zone of inhibition for each antibiotic according to the guidelines outlined in the “Performance Standards for Antimicrobial Susceptibility Testing”, CLSI M100 ED33:2023 [22].

#### 3.1.3. Machine Learning Datasets

In order to prepare the datasets for the implementation of machine learning algorithms, it was first necessary to pre-process the mass spectra obtained from the VITEK^®^ MS instrument. This instrument applies baseline removal, smoothing, and peak detection, resulting in a summary spectrum with approximately 200 *m/z* peaks distributed between 2000 Da and 12,000 Da. The VITEK^®^ MS instrument identifies microorganisms by analyzing ribosomal proteins within a specific mass range (commonly, 2000 to 20,000 Da). These masses are selected based on the characterization of ribosomal proteins, which are highly conserved and specific to each microbial species. To obtain fixed-length vectors, the mass spectra were discretized by a binning procedure, which consists of grouping the measured mass values into discrete ranges or “bins”, with the representative value being the average of the intensities within the bin. Binning has been applied in the range of 2000 to 10,000 Da with a bin size of 5 Da, which allows for a reasonable distribution of mass peaks without generating a vector that is too long to process computationally. In addition to spectral processing, each spectrum must be linked to its corresponding antibiotic resistance labels obtained from antimicrobial susceptibility testing. This linkage was achieved through a unique 8-digit numerical code that connects the laboratory report with the spectrum provided by the VITEK^®^ MS instrument. Figure 2 illustrates the number of samples included in this study, which consist of *Staphylococcus aureus*, *Escherichia coli*, and *Klebsiella pneumoniae*. These species were selected based on their relevance, the availability of samples, and their inclusion in the critical priority groups identified by the World Health Organization.

### 3.2. Machine Learning Classification

For the identification of antibiotic resistance, Support Vector Machine (SVM), Random Forest (RF), and Logistic Regression (LR) algorithms, which are among the most-utilized in the current literature [23], were considered. Additionally, CatBoost, a gradient-boosting algorithm known for its performance on tabular data [24], was implemented. In order to optimize the accuracy of the machine learning models used to predict antibiotic resistance, the Recursive Feature Elimination with Cross-Validation (RFECV) method was implemented. This approach enabled the automatic selection of the optimal set of relevant features for each of the evaluated models (SVM, LR, RF, and CatBoost). The RFECV process iteratively assessed the relevance of features using a base model, eliminating the least informative ones and validating the model’s performance through 10-fold stratified cross-validation. During this process, the Area Under the Precision–Recall Curve (AUPRC) was used as the optimized performance metric due to its focus on the positive class and its relevance in imbalanced data contexts. Subsequently, to optimize the configuration of these models, 10-fold cross-validation was used in a Bayesian hyperparameter search. During this search, the Area Under the Precision–Recall Curve (AUPRC) was optimized. This metric was calculated from precision and recall, focusing on the positive class, making it the ideal metric for evaluating binary classification models in imbalanced data environments.

In the Bayesian search for SVM, the values of ‘C’ and ‘gamma’ were optimized, considering a ‘rbf’ kernel. For Random Forest, the values of ‘n_estimators’, ‘min_samples_split’, and ‘max_features’ were optimized. In the case of Logistic Regression, the ‘penalty’ and ‘C’ parameters were optimized. Finally, for CatBoost, the parameters to be optimized were ‘iterations’, ‘depth’, ‘l2_leaf_reg’, and ‘learning_rate’. The models were implemented in Python using the scikit-learn library, while the hyperparameter search was performed using the ‘Skopt’ library. The final models underwent decision threshold calibration, as the default value of 0.5 in the scikit-learn library is suboptimal for imbalanced data environments.

Furthermore, another experiment was conducted to evaluate the effectiveness of the SMOTE (Synthetic Minority Oversampling Technique) on this type of data. All the classification models, SVM, RF, LR, CatBoost, and TL, were implemented using SMOTE. In this way, two separate experiments were performed: one without SMOTE and another incorporating the technique.

#### 3.2.1. Transfer Learning

To evaluate the performance of transfer learning compared to training from scratch using only local data, deep learning models reported in our previous study [25] were used—in detail, one model per bacterium. These models were previously trained on the massive public database of DRIAMS mass spectra [26]. The specific considerations for this work are detailed below:

- Selection of pre-trained models: Pre-trained models that addressed the same cases studied in this research were selected—specifically, for *Staphylococcus aureus* (oxacillin resistance), *Escherichia coli* (ceftriaxone resistance), and *Klebsiella pneumoniae* (ciprofloxacin resistance);
- Data alignment and preprocessing: Since the mass spectra used in the pre-trained models were obtained on a Bruker instrument, which provides a complete spectrum rather than just identified peaks, an alignment of the peaks reported by the VITEK^®^ MS instrument was performed. This alignment was performed using the mass range from the previous study as a reference [25]. Since the VITEK^®^ MS only reports about 200 peaks, the resulting spectrum was completed with zeros to adjust its length and resolution required by the pre-trained model;
- Transfer learning adjustments: Following the recommendations of the original deep learning study, both the convolutional and fully connected layers of the model were retrained using a learning rate of 0.0001. In addition, an “early stopping” technique was implemented to stop training when the AUPRC metric stopped improving, thus optimizing the fitting process;
- Cross-Validation: To ensure a fair comparison with the models trained from scratch, the same 10-fold cross-validation scheme used in the original machine learning models was applied, guaranteeing the consistency and robustness of the results obtained.

#### 3.2.2. Metrics

The models were implemented with a 10-fold cross-validation in order to avoid overfitting. Different metrics were calculated to evaluate model performance. Specifically, AUPRC, AUROC, balanced accuracy, and the F1 score were calculated. The AUPRC was calculated by integrating the precision-recall curve obtained by plotting precision (TP/(TP+FP)) against recall (TP/(TP+FN)). The AUROC measured the ability of the model to discriminate between positive and negative classes across all possible thresholds by plotting the true positive rate (recall) against the false positive rate (1 − specificity). Balanced accuracy was calculated as the average of sensitivity and specificity, adjusting for class imbalance by giving equal weight to each class. Finally, the F1 score, the harmonic mean of precision and recall, was particularly useful when the dataset was imbalanced, as it took into account both false positives and false negatives.

#### 3.2.3. Analysis of Features Contribution with Shapley Values

SHAP (SHapley Additive exPlanations) analysis was used to interpret the significance and impact of key features, specifically the (*m/z*) peaks, in predicting antibiotic resistance across bacterial species. SHAP is widely used for its versatility in explaining both classical machine learning models and neural networks. In this study, SHAP values were calculated for the CatBoost model using the TreeExplainer tool, which is tailored for decision tree-based algorithms.

## 4. Discussion

This research investigated the potential of MALDI-TOF mass spectrometry data in combination with machine learning algorithms to predict antimicrobial resistance in *Staphylococcus aureus*, *Escherichia coli*, and *Klebsiella pneumoniae* bacteria. The majority of preceding studies that have utilized these techniques were based on spectra obtained with Bruker equipment, which allows the complete spectrum to be exported for use in machine learning algorithms [23,25,26,27]. In contrast, this study examined the efficacy of mass spectra generated by VITEK^®^ MS in predicting resistance by applying a benchmarking of algorithms, including SVM, Logistic Regression, Random Forest, and CatBoost. Furthermore, we explore the application of models previously trained on MALDI-TOF data from Bruker [25] in order to assess the efficacy of transfer learning of these models to MALDI-TOF data from the VITEK^®^ MS instrument.

As shown in Table 1, tree-based algorithms, particularly the CatBoost model, provide an optimal approach for antimicrobial resistance identification. The gradient-boosting technique employed by the CatBoost model demonstrated particular efficacy in addressing the complex non-linear relationships inherent in mass spectra, outperforming all other algorithms across the evaluated metrics. These findings are consistent with those of previous studies that have also highlighted the superiority of gradient boosting models in heterogeneous data sets such as MALDI-TOF spectra [28,29].

Regarding transfer learning results, the performance proved to be unsatisfactory, with metrics underperforming compared to other models (Table 1). This may be due to differences in the resolution and characteristics of the spectra generated by the different instruments (Bruker and VITEK^®^ MS), suggesting that the pre-trained models require more specific adaptations to the local context to be effective. This finding emphasizes data standardization in future research aiming to apply transfer learning across different instruments. In this way, it is essential to explore new methodologies that enable transfer learning to integrate data from different mass spectrometry instruments to obtain adaptable models for diverse clinical settings and improve diagnostic accuracy for antimicrobial resistance.

In addition, the SMOTE technique did not improve the prediction of antibiotic resistance among *S. aureus*, *E. coli*, and *K. pneumoniae* (Table S1 in Supplementary Materials). Also, using SMOTE reduced performance by up to 10% for the AUROC and AUPRC metrics. For instance, in the case of *E. coli*, the AUROC decreased from 0.91 without SMOTE (Table 1) to 0.77 with SMOTE (Table S1 in Supplementary Materials).

In the analysis of potential biomarkers using SHAP values in the CatBoost model, several relevant peaks were identified as potential biomarkers for each bacterium under study. Specifically, for *S. aureus*, the peak at 3890 *m/z* was found to be associated with the SCCmec type II gene in the Jung-Min study [30]; the peak at 6550 *m/z* was associated with clone ST239, one of the most prevalent in hospital-acquired infections [31]; and the peak at 4810 *m/z* was related to clone ST45, a globally prevalent lineage of methicillin-resistant *S. aureus* (MRSA), which often causes severe invasive infections such as bacteremia [32].

Regarding the results from the UniProt search (Table 2), the RNA-metabolizing metallo-beta-lactamase protein (4890 *m/z*), commonly associated with ribonucleic acid metabolism, may play a role in resistance mechanisms, either through indirect interactions or as part of stress response pathways [33]. Additionally, the identification of the phage head–tail adapter protein (3120 *m/z*) suggests a possible involvement of prophages or horizontal gene transfer in modulating resistance phenotypes in *S. aureus* [34]. Furthermore, for *E. coli*, peaks at 8350 and 9700 *m/z* previously documented by Nakamura and colleagues [35] are associated with the ST131 lineage, one of the most predominant among multidrug-resistant *E. coli* isolates. Finally, for *K. pneumoniae*, no specific literature references were found for the reported peaks. However, according to UniProt, the dTDP-4-dehydrorhamnose 3,5-epimerase (6590 *m/z*), a protein involved in lipopolysaccharide biosynthesis, exhibited a strong association with antibiotic resistance [36]. Additionally, the identification of a transcriptional modulator YdgT (8305 *m/z*) further emphasizes the potential role of regulatory mechanisms in facilitating adaptive responses to antibiotic exposure [37].

These findings are especially significant from a clinical perspective given the growing prevalence of antibiotic-resistant pathogens in Chile and worldwide. The ability to predict antibiotic resistance quickly and accurately is crucial for optimizing treatments and improving patient outcomes. Integrating these tools into clinical practice could dramatically reduce the time needed to identify resistance, enabling more timely and appropriate therapies. Furthermore, infections caused by resistant microorganisms are closely associated with increased healthcare costs, higher mortality and morbidity rates, and prolonged hospital stays. These underscore the importance of effective antibiotic stewardship programs and infection control measures [38].

According to our findings, achieving timely and early treatment and reducing treatment duration for resistant pathogens represent significant leaps forward for public health. These are especially crucial for hospitalized patients with not only clinical risk factors but also social and economic factors, which may facilitate the transmission of antimicrobial resistance, likely due to limited access to timely and quality healthcare [39].

Finally, our results suggest that mass spectrum-based classification models have the potential to become a powerful tool in the fight against antimicrobial resistance. However, one of the main limitations identified in this study is the urgent need to standardize spectrum acquisition and preprocessing procedures. Such standardization is essential to enhance the generalization capabilities of the models, enabling their application across diverse clinical and geographical contexts, even when trained with data from different laboratories. In this way, our methodology presents promising avenues to combat the ongoing antimicrobial resistance crisis, in line with a One Health approach [40].

## 5. Conclusions

In conclusion, this study highlights the potential of integrating MALDI-TOF MS data with machine learning to predict antibiotic resistance in *S. aureus*, *E. coli*, and *K. pneumoniae*. Among the tested algorithms, the CatBoost model outperformed SVM, RF, RL, and transfer learning, achieving AUPRC and AUROC values of 0.73 or higher for all bacteria under study. While tree-based models demonstrated robust performance, transfer learning faced challenges due to instrument variability. Identified biomarkers provide valuable insights for future research, but standardizing data acquisition and optimizing transfer learning is essential to enhance generalizability and clinical applicability. These findings highlight the critical role of rapid and accurate resistance prediction in addressing the global challenge of antibiotic resistance.

## Figures and Tables

**Table 1:** The 10-fold cross-validation results for the models tested in each of the case studies.

	Algorithm	AUROC	AUPRC	B. Accuracy	F1 Score
*S. aureus* Oxacillin	SVM	0.78 ± 0.08	0.63 ± 0.11	0.64 ± 0.09	0.40 ± 0.21
	RF	0.86 ± 0.06	0.68 ± 0.09	0.74 ± 0.07	0.56 ± 0.11
	LR	0.81 ± 0.08	0.63 ± 0.09	0.73 ± 0.09	0.54 ± 0.12
	CatBoost	**0.86 ± 0.06**	**0.73 ± 0.09**	**0.77 ± 0.07**	**0.61 ± 0.12**
	TL	0.78 ± 0.09	0.56 ± 0.14	0.61 ± 0.09	0.34 ± 0.25
*E. coli* Ciprofloxacin	SVM	0.77 ± 0.09	0.74 ± 0.09	0.67 ± 0.09	0.58 ± 0.14
	RF	0.70 ± 0.07	0.77 ± 0.06	0.66 ± 0.07	0.63 ± 0.06
	LR	0.68 ± 0.11	0.66 ± 0.09	0.64 ± 0.11	0.58 ± 0.12
	CatBoost	**0.91 ± 0.07**	**0.91 ± 0.06**	**0.81 ± 0.08**	**0.78 ± 0.08**
	TL	0.70 ± 0.06	0.72 ± 0.14	0.68 ± 0.07	0.58 ± 0.11
*K. pneumoniae* Ciprofloxacin	SVM	0.65 ± 0.17	0.76 ± 0.11	0.62 ± 0.13	0.73 ± 0.12
	RF	0.69 ± 0.13	0.79 ± 0.09	0.58 ± 0.05	**0.78 ± 0.02**
	LR	0.60 ± 0.11	0.72 ± 0.07	0.57 ± 0.11	0.70 ± 0.08
	CatBoost	**0.73 ± 0.08**	**0.83 ± 0.07**	**0.65 ± 0.07**	0.78 ± 0.05
	TL	0.58 ± 0.12	0.71 ± 0.07	0.50 ± 0.09	0.76 ± 0.02

Values in bold represent the best performance for each case study.

**Table 2:** Set of the most important features and next significant protein biomarkers for the best performing models.

Bacteria	Antibiotic	Rank	Feature (Mass Da)	Uniprot Annotation	Uniprot ID
*S. aureus*	Oxacillin	1	3890	RNA-metabolizing metallo-beta-lactamase	A0A4P7P589
		2	5670	Uncharacterized protein	A0AAN0QQJ4
		3	2450	Nothing found	Nothing found
		4	6550	Membrane protein	A0AAE8TIP2
		5	3120	Phage head–tail adapter protein	A0A6G4JAS6
*E. coli*	Ciprofloxacin	1	5890	Inner membrane protein	A0A2X1JG16
		2	5230	Uncharacterized protein	A0A890DJW7
		3	3850	Phosphoenolpyruvate synthase	A0A3L9HBW1
		4	9225	DNA-binding protein HU-beta	P0ACF4
		5	8350	Plasmid maintenance protein CcdA	A0A074N0X8
*K. pneumoniae*	Ciprofloxacin	1	6590	dTDP-4-dehydrorhamnose 3,5-epimerase	A0A9Q4WW37
		2	5065	Mobile element protein	A0A2U8T1Q0
		3	6550	Uncharacterized protein	A0A0G2ST16
		4	4515	IS110 family transposase	A0A6M3YYK3
		5	8305	Transcription modulator YdgT	A0A0W8AUU2

## Data Availability

Available on request from the corresponding author.

## Supplementary Materials

The following supporting information can be downloaded at: https://www.mdpi.com/article/10.3390/ijms1010000/s1.
