# Supplementary material for: Integrating Machine Learning with MALDI-TOF Mass Spectrometry for Rapid and Accurate Antimicrobial Resistance Detection in Clinical Pathogens
Source: Int J Mol Sci. 2025 Jan 28;26(3):1140. doi: 10.3390/ijms26031140 (PMC11817502; doi:10.3390/ijms26031140)
Supplement: Supplementary file 1 [file ijms-26-01140-s001.zip › ijms-3368007-supplementary.pdf]

Integrating Machine Learning with MALDI-TOF Mass Spectrometry for Rapid and Accurate Antimicrobial Resistance Detection in Clinical Pathogens

Supplementary Material

November 27, 2024

Oversampling implementation

Table S1

To deal with the class imbalance, the SMOTE oversampling technique from the 'imbalanced learn' Python library has been implemented. As can be seen in Table S1, the trend of the results is maintained, with CatBoost being the algorithm with the best results, but these turn out to be lower than the results without oversampling.

	Algorithm	Auroc	Auprc	B. accuracy	F1
S. aureus Oxacillin	SVM	0.67 ± 0.02	0.46 ± 0.04	0.61 ± 0.02	0.36 ± 0.04
	RF	0.80 ± 0.01	0.62 ± 0.02	0.64 ± 0.01	0.42 ± 0.03
	LR	0.74 ± 0.01	0.53 ± 0.01	0.65 ± 0.00	0.41 ± 0.01
	CatBoost	**0.80 ± 0.01**	**0.65 ± 0.02**	**0.66 ± 0.02**	**0.46 ± 0.03**
	TL	0.76 ± 0.09	0.52 ± 0.08	0.66 ± 0.08	0.44 ± 0.10
E. coli Ciprofloxacin	SVM	0.70 ± 0.08	0.67 ± 0.09	0.66 ± 0.07	0.60 ± 0.12
	RF	0.75 ± 0.01	0.76 ± 0.01	0.70 ± 0.01	0.64 ± 0.02
	LR	0.68 ± 0.00	0.65 ± 0.01	0.62 ± 0.00	0.57 ± 0.01
	CatBoost	**0.77 ± 0.01**	**0.77 ± 0.02**	**0.70 ± 0.01**	**0.64 ± 0.02**
	TL	0.72 ± 0.10	0.71 ± 0.09	0.65 ± 0.11	0.59 ± 0.16
K. pneumoniae Ciprofloxacin	SVM	0.56 ± 0.03	0.69 ± 0.02	0.54 ± 0.03	0.63 ± 0.08
	RF	**0.68 ± 0.02**	0.77 ± 0.01	**0.65 ± 0.02**	**0.75 ± 0.02**
	LR	0.59 ± 0.02	0.71 ± 0.01	0.56 ± 0.01	0.64 ± 0.01
	CatBoost	0.67 ± 0.02	**0.78 ± 0.02**	0.62 ± 0.02	0.72 ± 0.02
	TL	0.66 ± 0.09	0.76 ± 0.06	0.56 ± 0.11	0.67 ± 0.18

Table 1: Results of the 10-fold cross-validation for the models tested in each of the case studies when the SMOTE technique is used to deal with class imbalance.
